# Predictive factors for segmental lordosis angle restoration in oblique lumbar interbody fusion at L4/5

**DOI:** 10.1186/s12893-025-03401-x

**Published:** 2025-12-17

**Authors:** Ji-Le Jiang, Tenghui Ge, Ronghui Cai, Guanqing Li, Jintao Ao, Jingye Wu, Zhao Lang, Yuqing Sun

**Affiliations:** Department of Spine Surgery, Beijing Jishuitan Hospital, Capital Medical University, No. 31, Xinjiekou East Street, Xicheng District, Beijing, 100035 People’s Republic of China

**Keywords:** Oblique lumber interbody fusion, Segmental lordosis angle, Radiological outcomes, Cage subsidence, Cage position

## Abstract

**Study design:**

Retrospective study.

**Objectives:**

To identify the factors that determine segmental lordosis angle (SLA) at L4/5 after oblique lumber interbody fusion (OLIF).

**Methods:**

A total of 65 patients who underwent single-level L4/5 OLIF (6°-lordotic cage) with posterior pedicle screw fixation without posterior osteotomy for lumbar degenerative disease were analyzed. The SLA was measured preoperatively (pre), postoperatively (post), and at the last follow-up (last) (at least 12 months) on the midline sagittal CT views. Demographics, surgical, and radiological factors were included. Clinical outcomes (ODI and VAS) were assessed.

**Results:**

The mean preoperative SLA at L4/5 increased from 7.1 ± 3.9° preoperatively to 9.8 ± 3.4° postoperatively (*P* < 0.001) and was maintained as 9.1 ± 3.5° at the last follow-up (*P* < 0.001). Multiple regression analysis revealed that Pre-SLA, cage position and cage subsidence were predictors of Last-SLA. Small-angle group (Pre-SLA < 6°) showed a stronger capacity in the increase of SLA (Pre-SLA – Post-SLA) and change of SLA (Last-SLA – Pre-SLA) than large-angle group (Pre-SLA ≥ 6°). Anterior cage position is associated with the more increase of SLA than posterior cage position. Cage subsidence led to approximately 3° in decrease of SLA (Last-SLA – Post-SLA). No significant correlation was observed between Last-SLA and clinical outcomes.

**Conclusion:**

The Last-SLA is strongly associated with the Pre-SLA condition and OLIF has a higher capability to restore SLA in patients with a smaller preoperative SLA. To achieve a greater SLA, surgeons should try to insert cage in the anterior disc space and reduce the occurrence of cage subsidence.

## Introduction

Proper lumbar lordosis restoration has been considered as a critical factor in achieving a well-balanced spine and reducing postoperative complications [1]. Approximately two-thirds of global lumbar lordosis occurred at the distal lumbar lordosis including L4/5 and L5-S1, which determines the shape of lumbar lordosis [2, 3]. L4/5 segmental lordosis angle (SLA) in asymptomatic adults has been documented by several previous studies as a mean angle of 8–13° [4–7].

Adequate restoration of segmental lordosis angle is important to decrease adjacent segment degeneration and achieve appropriate sagittal balance in patients undergoing lumbar fixation surgery even in short-level spinal surgery [8, 9]. Oblique lumbar interbody fusion (OLIF) is a recently developed minimally invasive surgery to restore lumbar lordosis by lordotic cages at each level, which has been demonstrated to be effective for some correction in degenerative lumbar diseases with less blood loss and lower rates of morbidity than traditional osteotomy techniques [10, 11].

Although OLIF with pedicle screw fixation has been reported favorable radiological outcomes for degenerative lumbar diseases [12, 13], it is poorly understood how much SLA can be obtained or which factors determine the SLA at the last follow-up. The current study aimed to identify the factors that can contribute to greater SLA after OLIF at L4/5.

## Materials and methods

### Patient selection

This was a retrospective study of patients who underwent OLIF from October 2017 to October 2019 in our institution. The study was approved by the ethical committee of the authors’ hospital. Inclusion criteria included a single-level interbody fusion of OLIF with bilateral pedicle screw fixation for L4/5 degenerative lumbar diseases and follow-up for more than 12 months. Exclusion criteria included multi-level fusions, the inclusion of spinal osteotomies, and abnormal endplates, such as previous fracture, previous lumbar fusion, infections, tumors, or Schmorl’s nodes.

### Operative technique

OLIF surgeries were performed by the standard procedure [14]. The patient was placed in the right lateral decubitus position. The OLIF25 cages (Clydesdale Spinal System, Medtronic) were 6° in lordotic angle, 18 mm in width, 45, 50, 55, or 60 mm in length, and 10, 12–14 mm in height. The appropriate size of cage filled with demineralized bone matrix and allogeneic bone material was inserted in the optimal position. Then the patient was prone positioned and the bilateral pedicle screw fixation was performed on each patient. All patients did not perform facet osteotomies. Laminar fenestration for direct decompression was performed for some patients.

### SLA and related factors

The primary outcome of this study was the SLA at the last follow-up. The SLA was measured on the midline sagittal CT views preoperatively (pre), postoperatively (post), and at the last follow-up (last). The SLAs were independently measured and assessed by two observers who received training of measurement. The average value of two measurements was calculated and used for statistical analysis.

**Fig. 1:**
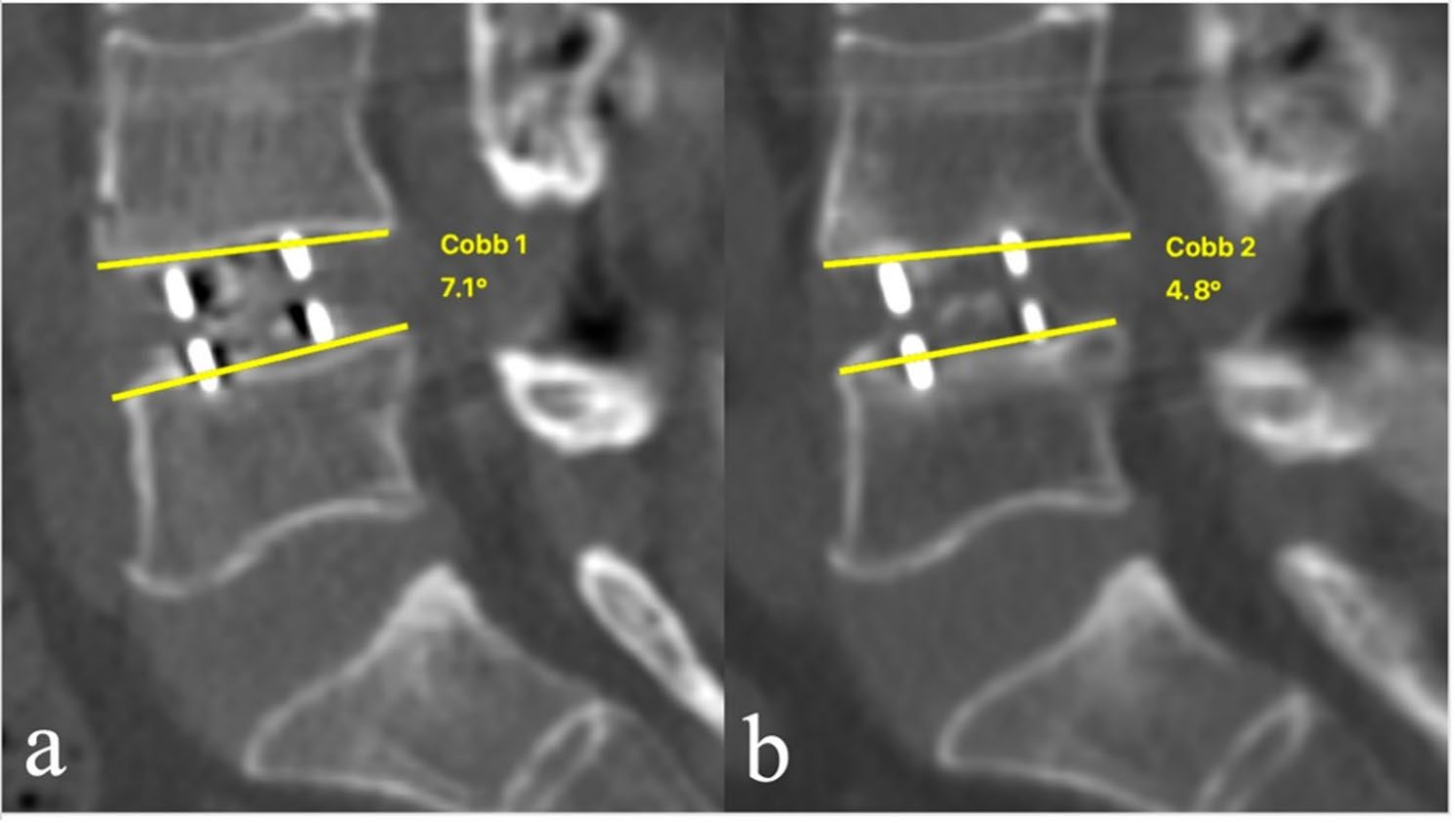
Radiological measurement. **a** Disc height (DH) and Segmental lordosis angle (SLA); **b** Cage position (CageP)

Related data included demographics, surgical, and radiological factors. Demographic factors included age, gender, body mass index (BMI), bone mineral density (BMD), smokers, and diabetes. BMD was calculated as the average among lumbar spine (L1–L3) trabecular volumetric BMD by quantitative computed tomography. Surgical factors included cage parameters (height and length) and decompression methods. Radiological factors included segmental lordosis angle (SLA), disc height (DH), cage position (CageP), spondylolisthesis, facet osteoarthritis, Modic type 2 change, disc height gap (CageP–DH), intraoperative endplate injury, cage subsidence and fusion status. SLA, DH and CageP were measured on the midline sagittal CT views (Fig. 1). SLA was defined as the angle between the upper endplate and the lower endplate in the disc space. DH was defined as the shortest distance between the midpoint of L5 upper endplate and L4 lower endplate. CageP was defined as the ratio between the center of the cage and anterior edge of the upper endplate of the inferior vertebra to the length of the upper endplate of the inferior vertebra. Spondylolisthesis was defined as ≥ 3 mm vertebral slipping. Modic type 2 change was assessed on the T1- and T2-weighted sagittal magnetic resonance images. Facet osteoarthritis was assessed by Pathrea-CT classifications [15], including Grade I (Normal), Grade II (Mild), Grade III (Moderate), and Grade IV (Severe). Fusion was defined as the presence of bridging interbody trabecular bone. Intraoperative endplate injury was defined as cage breaching into the adjacent cortical endplate more than 2 mm. Cage subsidence was defined as the disc height loss of more than 2 mm between postoperatively and at the last follow-up [16].

Clinical outcomes were evaluated by the visual analog scale for back pain (VAS-B) and leg pain (VAS-L), and the Oswestry disability index (ODI) before surgery and after at least 12 months.

### Statistical analysis

All statistical analyses were performed using SPSS software version 26.0 (SPSS, Chicago, IL). The interobserver reliability was assessed by intra-class correlations coefficients (ICCs) for SLA measurements. Associations between the Last-SLA and each categorical variable were assessed using independent sample *t*-test and a one-way analysis of variance test. Correlations between the Last-SLA and each continuous variable were accessed using Pearson correlation coefficients. Variables with P-value < 0.1 in univariate analyses were included in multivariate linear regression analysis and the collinearity was assessed with the variance inflation factor (VIF). Independent sample *t*-test was performed in subgroup analysis. A correlation between the Last-SLA and clinical outcomes was verified using Pearson correlation coefficients. Statistical significance was accepted at P-value < 0.05.

## Results

### Measurement accuracy and change in SLA after OLIF

The ICC for all SLA measurements were 0.872, as ICC value was divided as poor (0–0.39), fair to good (0.4–0.74), or excellent (0.75–1). Angular measurements were deemed accurate and reproducible. The SLA increased from 7.1 ± 3.9° preoperatively to 9.8 ± 3.4° postoperatively (*P* < 0.001) and was maintained as 9.1 ± 3.5° at the last follow-up (*P* < 0.001).

### Demographics and related factors

The study population consisted of 65 patients (42 women and 23 men; mean age, 58.8 ± 10.1 years; age range, 33–80 years). The mean follow-up time was 20.6 ± 8.3 months (range, 12–40). The mean body mass index was 26.2 ± 3.3 kg/m^2^ (range 20.8–35.2). The mean bone mineral density was 101.9 ± 40.0 mg/cm^3^ (range 32.4–200.1). The number of smokers was 7 (10.8%) and the number of patients with diabetes was 9 (13.8%).

Indirect decompression in OLIF was performed in 35 (53.8%) cases, and direct decompression with fenestration in 30 (46.2%) cases. The cage height was 10 mm in 5 (7.7%), 12 mm in 47 (72.3%), and 14 mm in 13 (20.0%) cases. The cage length was 45 mm in 3 (4.6%), 50 mm in 29 (44.6%), 55 mm in 29 (44.6%) and 60 mm in 4 (6.2%) cases.

The preoperative SLA and DH were 7.1 ± 3.9° (range − 8.7–14.7) and 8.5 ± 2.4 mm (range 3.5–13.7). Spondylolisthesis and Modic type 2 change were found in 35 (53.8%) and 9 (13.8%) patients. The facet joint osteoarthritis was Grade I in 5 (7.7%), Grade II in 18 (27.7%), Grade III in 18 (27.7%), and Grade IV in 24 (36.9%) patients. The cage position was 47.3 ± 8.1% (range 28.8–63.4) postoperatively. Intraoperative endplate injury was found in 14 (21.5%) patients. Cage subsidence at the last follow-up was found in 12 (22.6%) patients. Fusion at the last follow-up was found in 61 (93.8%) patients.

### Effects of various factors on Last-SLA

The results of the univariate analysis showed that age, Pre-SLA, cage position, intraoperative endplate injury, cage subsidence and cage height were factors associated with Last-SLA (Table 1). The multiple linear regression analysis showed that Pre-SLA, cage position, and cage subsidence were the independent significant predictors of Last-SLA, and the adjusted coefficient of determination (adjusted R^2^) was 0.550 (Table 2). There was no significant multicollinearity among the independent variables.

**Table 1:** Association between factors and Last-SLA

	Patients (*n* = 65)	Last-SLA	Correlation coefficient	*P*-value
Demographics factors				
Age (years)	58.8 ± 10.1		0.249	0.046†*
Gender				
Male	23	8.8 ± 2.8		0.704
Female	42	9.2 ± 4.0		
Body mass index (kg/m^2^)	26.2 ± 3.3		0.032	0.799
Bone mineral density (mg/cm^3^)	101.9 ± 40.0		-0.055	0.664
Follow-up time (months)	20.6 ± 8.3		-0.136	0.282
Smokers				
Yes	7	8.7 ± 3.4		0.777
No	58	9.1 ± 3.6		
Diabetes				
Yes	9	9.8 ± 3.6		0.472
No	56	8.9 ± 3.5		
Surgical factors				
Decompression method				
Indirect decompression	35	9.7 ± 3.2		0.140
Direct decompression with fenestration	30	8.3 ± 3.8		
Cage height				
10 mm	5	11.7 ± 3.5		0.075†
12 mm	47	8.5 ± 3.4		
14 mm	13	10.1 ± 3.5		
Cage length				
45 mm	3	7.9 ± 9.4		0.808
50 mm	29	9.1 ± 2.8		
55 mm	29	8.9 ± 3.5		
60 mm	4	10.4 ± 3.9		
Radiological factors				
Preoperative parameters				
Preoperative SLA (°)	7.1 ± 3.9		0.622	< 0.001†*
Preoperative disc height (mm)	8.5 ± 2.4		-0.096	0.449
Spondylolisthesis				
Yes	35	9.3 ± 4.4		0.486
No	30	8.7 ± 2.2		
Facet osteoarthritis				
Grade I	5	8.0 ± 2.0		0.522
Grade II	18	9.5 ± 3.2		
Grade III	18	8.2 ± 3.2		
Grade IV	24	9.6 ± 4.2		
Modic type 2 change				
Yes	9	10.5 ± 3.5		0.181
No	56	8.8 ± 3.5		
Postoperative parameters				
Cage position (%)	47.3 ± 8.1		-0.389	0.001†*
Disc height gap (mm)	3.7 ± 2.3		0.095	0.452
Intraoperative endplate injury				
Yes	14	6.4 ± 3.5		0.001†*
No	51	9.8 ± 3.2		
Cage subsidence				
Yes	12	7.3 ± 4.6		0.062†
No	53	9.4 ± 3.2		
Fusion status				
Yes	61	9.0 ± 3.5		0.489
No	4	10.3 ± 5.0		

† means *P*-value < 0.1; * means *P*-value < 0.05

**Table 2:** Multivariate linear regression analysis

Factors	Unstandardized		Standardized β	t	P	VIF	Adjusted R^2^
	B	SE					
(Constant)	12.846	2.971	-	4.323	< 0.001*	-	0.550
Age	0.015	0.032	0.043	0.482	0.631	1.146	
Preoperative SLA	0.507	0.087	0.551	5.807	< 0.001*	1.282	
Cage position	-0.156	0.038	-0.356	-4.088	< 0.001*	1.082	
Intraoperative endplate injury	-0.613	0.841	-0.072	-0.728	0.469	1.379	
Cage subsidence	-1.934	0.863	-0.214	-2.241	0.029*	1.292	
Cage height (12 mm)	-0.680	1.170	-0.087	-0.581	0.563	3.160	
Cage height (14 mm)	0.430	1.323	0.049	0.325	0.747	3.231	

∗ means statistically significant (*P*-value < 0.05)

### Subanalysis based on Pre-SLA

**Fig. 2:**
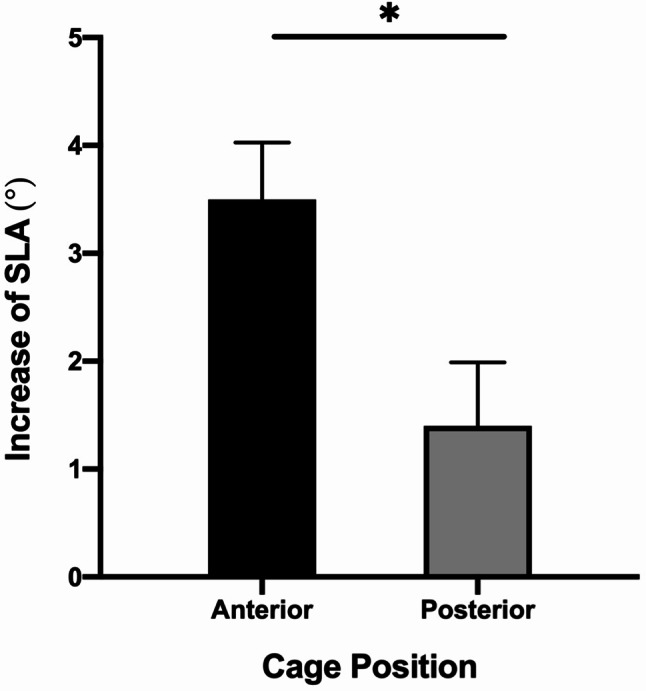
Representative cases in respect to Pre-SLA in large-angle group (Pre-SLA ≥ 6°) (**a** **b** **c**) and small-angle group (Pre-SLA < 6°) (**e** **f** **g**) preoperatively, postoperatively, and the last follow-up

As the lordotic angle of cage applied in this study was 6°, we defined small angle as Pre-SLA < 6° and large angle as Pre-SLA ≥ 6°. Therefore, we further analyzed the effects of Pre-SLA on the increase of SLA (Pre-SLA – Post-SLA), decrease of SLA (Last-SLA – Post-SLA) and change of SLA (Last-SLA – Pre-SLA). Small-angle group showed a stronger capacity in the increase of SLA and change of SLA than large-angle group (*P* < 0.001 for both) (Table 3; Fig. 2).

**Table 3:** Subanalysis based on Pre-SLA

SLA	All (*N* = 65)	Subgroup		
		Low angle group Pre-SLA < 6° (*N* = 21)	High angle group Pre-SLA ≥ 6° (*N* = 44)	*P*-value
Increase of SLA (Pre-SLA – Post-SLA)	2.7 ± 3.3	4.9 ± 3.1	1.6 ± 3.0	< 0.001*
Decrease of SLA (Last-SLA – Post-SLA)	0.7 ± 1.6	0.9 ± 1.6	0.6 ± 1.6	0.475
Change of SLA (Last-SLA – Pre-SLA)	1.9 ± 3.2	4.0 ± 2.6	1.0 ± 3.1	< 0.001*

∗ means statistically significant (P-value < 0.05)

### Subanalysis based on cage position

**Fig. 3:**
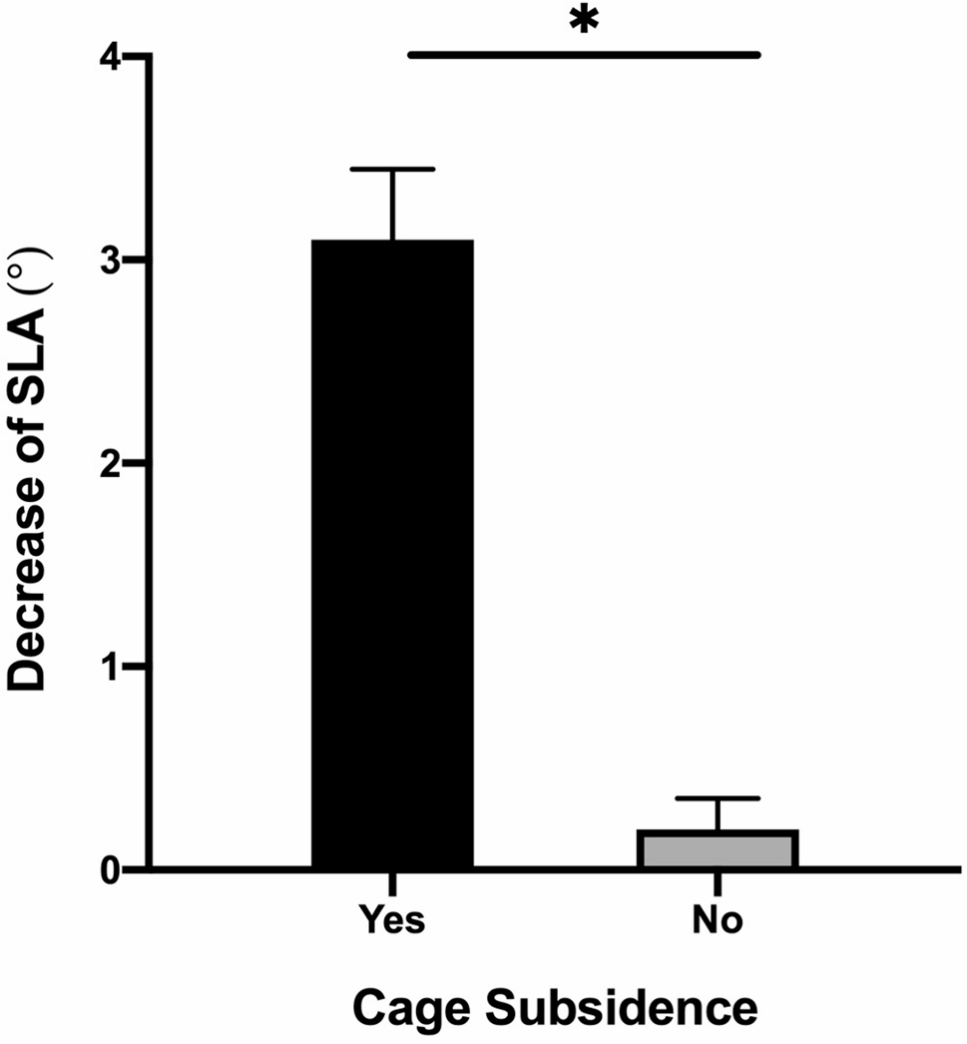
The increase of SLA was significantly different between anterior (Cage-*P* < 50%) and posterior (Cage-*P* ≥ 50%) cage positions

**Fig. 4:**
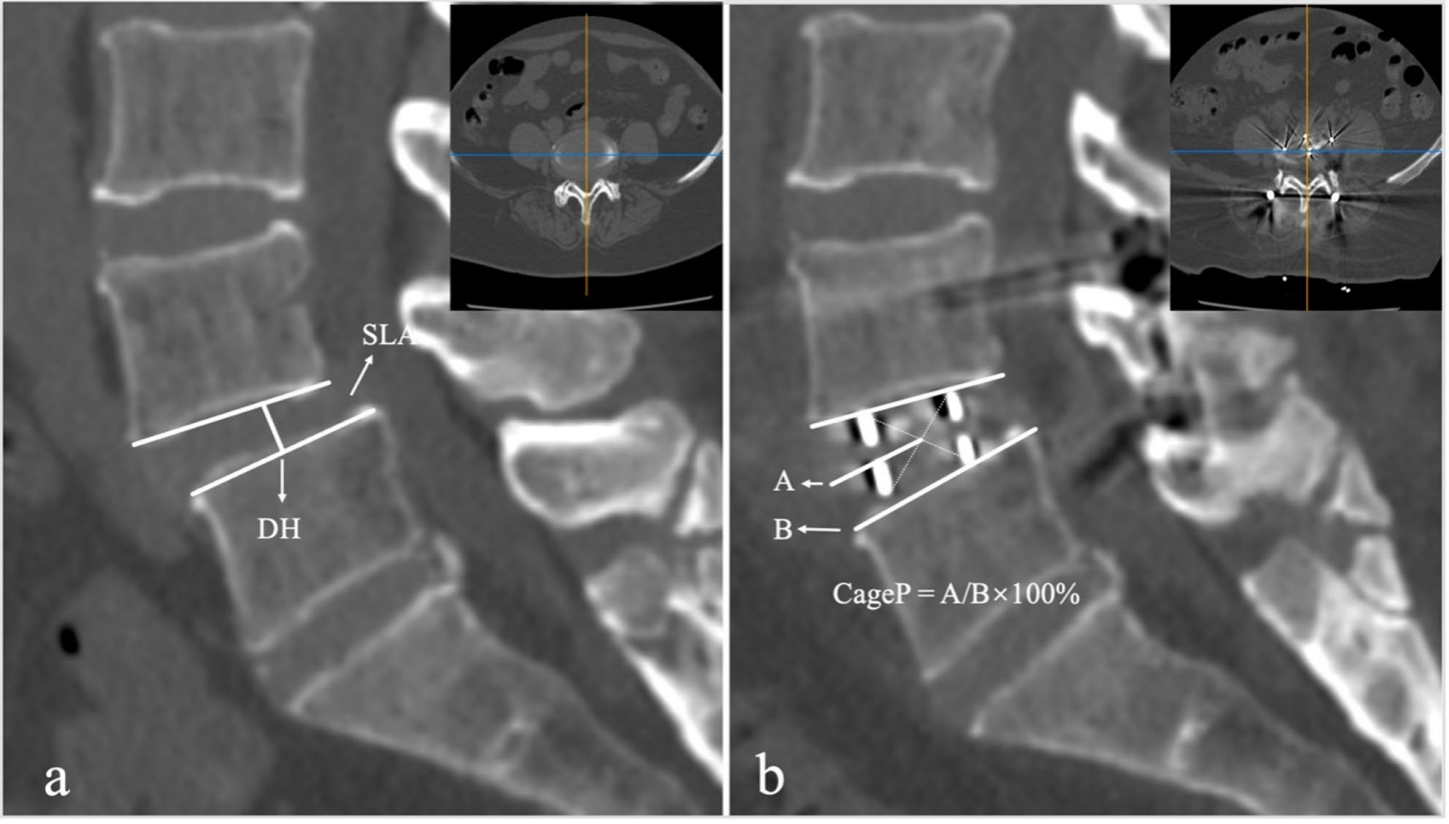
Representative cases in respect to cage position in anterior cage position (Cage-*P* < 50%)(a, b) and posterior cage position (Cage-*P* ≥ 50%) (c, d)preoperatively and postoperatively

The effect of cage position on the increase of SLA (Pre-SLA – Post-SLA) was analyzed. Anterior cage position was defined as Cage-*P* < 50% and posterior cage position was defined as Cage-*P* ≥ 50%. Anterior cage position is associated with a more increase of SLA than posterior cage position (3.5 ± 3.3° vs. 1.4 ± 3.0°, *P* = 0.010) (Figs. 3 and 4).

### Subanalysis based on cage subsidence

**Fig. 5:**
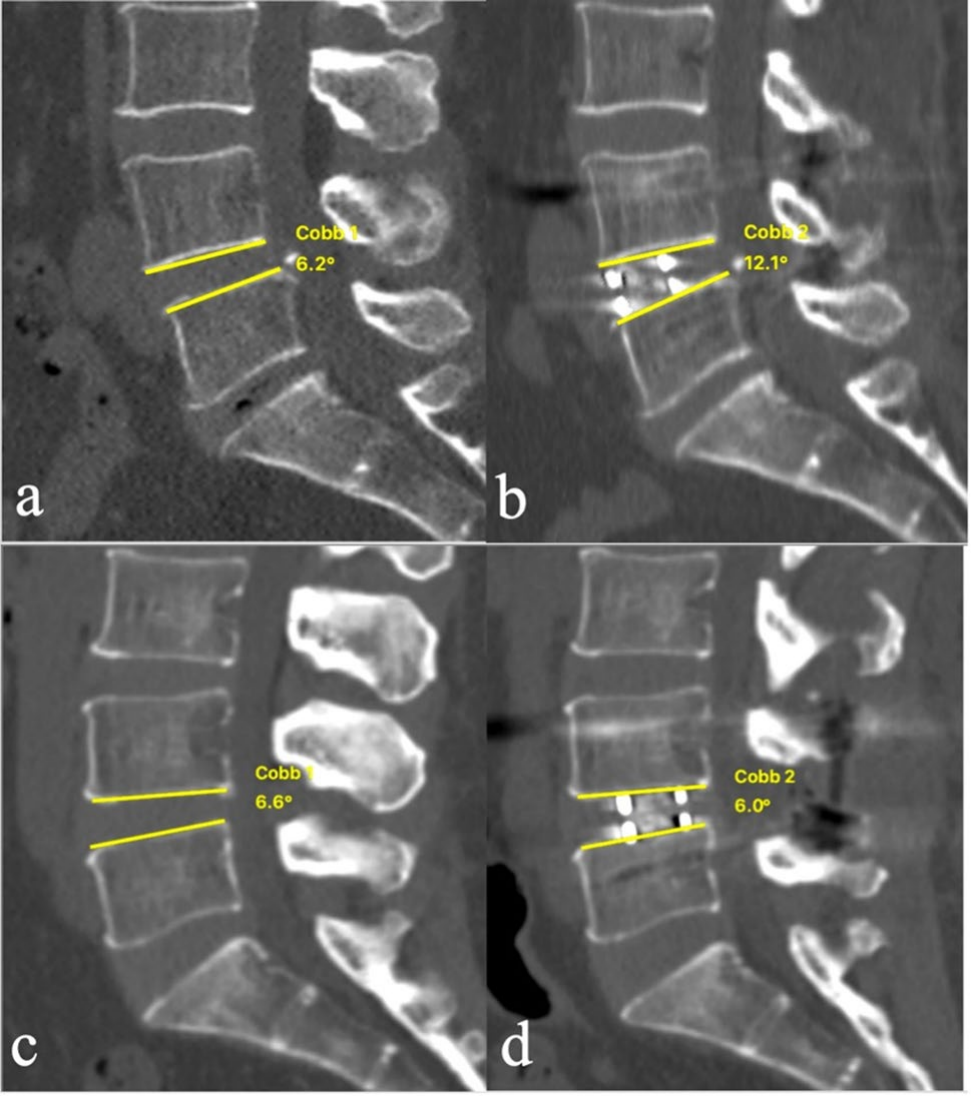
The decrease of SLA was significantly different between cage subsidence and no cage subsidence

**Fig. 6:**
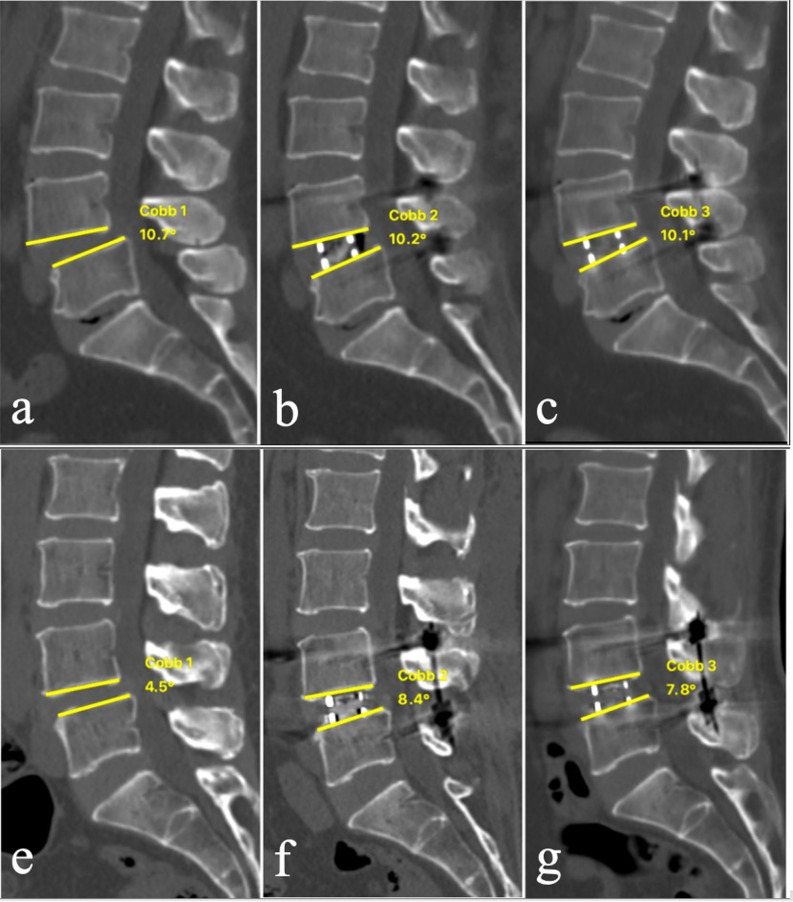
Representative cases in respect to cage subsidence postoperatively(a) and at the last follow-up(b)

The effect of cage subsidence on the decrease of SLA (Post-SLA **–** Last-SLA) was analyzed. Cage subsidence led to a significantly more loss of SLA in operative levels than those without cage subsidence (3.1°±1.2 vs. 0.2 ± 1.1°, *P* < 0.001) (Figs. 5 and 6).

### Clinical outcomes

The average VAS-B before surgery was 4.6 ± 3.0 and at last follow-up was 1.7 ± 1.9. The average VAS-L before surgery was 6.2 ± 2.3 and at last follow-up was 1.6 ± 2.0. The average ODI before surgery was 49.0 ± 20.8% and at last follow-up was 10.0 ± 10.8%. No correlations were observed between Last-SLA and VAS-B (*r* = 0.026), VAS-L (*r* = -0.088), ODI (*r* = 0.055) at last follow-up. No patients developed symptomatic adjacent segment disease.

## Discussion

Compared with posterior or transforaminal lumbar interbody fusion, one common advantage of the OLIF is its ability to restore segmental lordosis angle [13, 17]. It is known that numerous factors influence SLA restoration in OLIF, including cage dimension, cage position, anterior column release, and posterior osteotomies [18–20]. Although many of these factors make intuitive sense, it has not been clarified yet that how to achieve proper SLA at the last follow-up. The radiological parameters were measured by midline sagittal CT views because CT-based measurements might be considered as more accurate measurements to assess segmental alignment than plain radiographs. Our results showed that the Last-SLA in L4/5 levels was 9.1 ± 3.5°, which was basically consistent with the mean normal value in asymptomatic adult subjects (Approximately 10°) [4–7]. And the change of SLA in L4/5 levels was 1.9 ± 3.2°, which is comparable to the previous studies (1.6°−5.7°) [12, 13, 17, 19, 21–23].

Recent studies have advanced our understanding of harmonious sagittal alignment by reporting mean segmental lumbar lordosis values based on pelvic incidence (PI) [24]. For individuals with a PI ranging between 40 ° and 70 °, the typical segmental lordosis at L4/5 is approximately 15 °, measured across both vertebral bodies and the intervertebral disc. These values can serve as a general reference for surgical planning. The work of Pesenti et al. [25] has helped clarify the regional distribution of lumbar lordosis in asymptomatic adults, observing that PI predominantly influences proximal lumbar lordosis, while the lordosis across the L4–S1 segment remains relatively constant, averaging around 36 °. Clinical evidence indicates that applying such segmental angle parameters in degenerative spinal conditions can effectively reduce the risks of postoperative malalignment, adjacent segment disease, and revision surgery [24].

In our study, we found that Last-SLA in OLIF at L4/5 was associated with Pre-SLA, cage position and cage subsidence. Among these, Pre-SLA was identified as the primary factor influencing Last-SLA, suggesting that a larger Pre-SLA may facilitate achieving a sufficient Last-SLA, whereas a smaller Pre-SLA may present a greater challenge. Otsuki et al. [18] also reported that Pre-SLA was significantly associated with the SLA restoration in lateral lumbar interbody fusion. Interestingly, the magnitude of SLA increase was negatively correlated with Pre-SLA. This inverse relationship suggests that a greater degree of SLA improvement is more readily achievable when the Pre-SLA is small.

Cage position is considered as an important predictive factor for greater Last-SLA, which can be handled by surgeons themselves. Anterior cage placement resulted in greater SLA than posterior cage placement, which was consistent with the previous studies [18, 22, 23]. While anterior cage placement can improve SLA effectively by tightening the anterior longitudinal ligament and impacting the facet joint, it is not without disadvantages, as it might increase the risk of cage subsidence and potential failure of indirect neural decompression. Yasuhiro et al. [23] reported that anterior cage placement was associated with a high incidence of postoperative endplate injury and large lordosis correction. The change of posterior disc height was thought to be associated with the indirect decompression effect and posterior cage placement was beneficial to decompressing the central and foraminal canal [26]. Surgeons performing OLIF should make a balance between the amount of lordosis restoration needed and the necessity of indirect neural decompression.

There is a negative association between cage subsidence and Last-SLA. In our series, decrease of SLA by cage subsidence was approximately 3°. Cage subsidence was a spontaneous reaction between the cage and endplate, which occurs gradually over the postoperative course [26]. A previous cadaveric study showed that endplate strength in posterior regions was stronger than those in anterior regions [27]. Grant et al. [28] found that the upper endplate was mechanically weaker than the lower endplate. Therefore, cage subsidence is common at the anterior corner of the cage in the upper endplates of the caudal vertebra, which resulted in a loss of SLA.

In the current study, OLIF had a relatively small correction angle of 2.7º postoperatively and 1.9 º at the last follow-up at L4/5 levels considering the 6º lordotic angle of the cage in OLIF procedure. Although the greater lordotic cage angle theoretically resulted in achievement of greater SLA following OLIF for degenerative lumbar conditions, prior research found that the lordotic cage angle was related to SLA only once the cage lordotic angle increased to 15° [19]. In order to attain maximum SLA, it is important to insert cage in the anterior disc space and reduce the occurrence of cage subsidence. Our results also found that if the Pre-SLA is small, the increase of SLA is large but Last-SLA might be relatively small; if Pre-SLA is large, the increase of SLA is small but Last-SLA might be relatively large. Moreover, the anatomical limitations including anterior longitudinal ligament and bilateral facet joints had a “ceiling effect” on the ability of lordosis correction in OLIF procedure [29]. Resection of the anterior longitudinal ligament and posterior structure can result in more segmental lordosis. If surgeons want to increase greater lordosis through OLIF, they may need to release the restricting anatomical structures and insert the cage in the anterior disc space.

This study has several limitations that should be considered when interpreting the results. First, the retrospective design and relatively small sample size may introduce selection bias and limit the generalizability of our findings. Second, the evaluation of cage lordotic angle was restricted by the exclusive use of 6° cages in our institutional cohort. Third, while CT measurements in the supine position provide superior accuracy for assessing segmental parameters, they may not adequately represent spinal alignment under functional standing conditions. Furthermore, heterogeneity in follow-up durations across patients may affect the interpretation of long-term outcomes. Future prospective studies with larger cohorts, standardized follow-up protocols, and comparative assessments of different cage angles are warranted to validate and extend these long-term findings.

## Conclusion

A strong association was observed between the Last-SLA and Pre-SLA at L4/5 following OLIF. Specifically, the procedure demonstrated a greater potential for restoring lordosis in patients with a lower Pre-SLA. To achieve optimal segmental lordosis, surgeons should aim for anterior cage placement within the disc space and employ strategies to mitigate the risk of cage subsidence.

## Data Availability

The data used to support the findings of this study are available from the corresponding author upon request.

## Abbreviations

OLIF: Oblique Lumbar Interbody Fusion
SLA: Segmental Lordosis Angle
CT: Computed Tomography
ODI: Oswestry Disability Index
VAS-B: Visual Analog Scale for Back pain
VAS-L: Visual Analog Scale for Leg pain
DH: Disc Height
CageP: Cage Position
CageP-DH: Disc Height Gap
BMD: Bone Mineral Density
Q, DXA: Dual-energy X-ray Absorptiometry
BMI: Body Mass Index
DH: Disc Height
ICCs: Intra-class Correlations Coefficients
VIF: Variance Inflation Factor
